# Beyond the BMI Paradox: Unraveling the Cellular and Molecular Determinants of Metabolic Health in Obesity

**DOI:** 10.3390/biom15091278

**Published:** 2025-09-03

**Authors:** Kyoichiro Tsuchiya, Takahiro Tsutsumi

**Affiliations:** Department of Diabetes and Endocrinology, Graduate School of Interdisciplinary Research, Faculty of Medicine, University of Yamanashi, Chuo 409-3821, Japan

**Keywords:** obesity phenotypes, insulin sensitivity, fat distribution, adipose tissue function, cardiometabolic risk, inflammation, obesity paradox, personalized medicine

## Abstract

Obesity has traditionally been considered a major risk factor for numerous metabolic disorders and diseases. However, a subset of individuals with obesity, classified as having “metabolically healthy obesity” (MHO), display relatively normal metabolic parameters despite excess adiposity. This review critically examines the current knowledge surrounding MHO, including its various definitions, prevalence, clinical characteristics, contributing factors, and long-term outcomes. While MHO carries lower health risks compared to metabolically unhealthy obesity (MUO), evidence consistently demonstrates increased disease risk compared to metabolically healthy normal-weight individuals, particularly for type 2 diabetes, cardiovascular disease, chronic kidney disease, and certain cancers. MHO prevalence ranges from 10 to 30% among individuals with obesity globally, varying by sex, age, BMI, and ethnicity. Multiple factors contribute to the MHO phenotype, including beneficial adipose tissue distribution patterns, enhanced adipocyte function, favorable genetic profiles, and lifestyle factors. Recent single-cell transcriptomic analyses have identified specific cell populations, particularly mesothelial cells, as key drivers of metabolic health in visceral adipose tissue. The discovery of persistent epigenetic memory of obesity provides molecular evidence for why MHO often represents a transient state, with many individuals progressing to MUO over time. Emerging evidence also reveals differential therapeutic responses to GLP-1 receptor agonists between MHO and MUO phenotypes, highlighting the need for precision medicine approaches. The concept of MHO has important clinical implications for risk stratification and personalized treatment approaches. This review synthesizes current evidence while highlighting knowledge gaps and future research directions in this rapidly evolving field.

## 1. Introduction

Obesity has reached pandemic proportions, with current projections suggesting that over half of the world’s population will have overweight or obesity by 2035 [1]. Recent data from the 2024 National Health and Nutrition Examination Survey indicates that obesity prevalence among US adults has reached 40.3% during August 2021–August 2023, emphasizing the urgency of understanding this condition [2]. The World Health Organization defines obesity as abnormal or excessive fat accumulation that presents a risk to health, typically indicated by a body mass index (BMI) of 30 kg/m^2^ or higher in Western populations and 25 kg/m^2^ or higher in Asian populations [3]. Obesity is recognized as a major risk factor for numerous conditions including insulin resistance, type 2 diabetes, dyslipidemia, hypertension, cardiovascular disease, chronic kidney disease, and various cancers [3].

However, the relationship between excess adiposity and metabolic complications is not uniform. Since the early 2000s, clinical observations have highlighted a unique subset of obese individuals with obesity who display relatively normal metabolic parameters despite excess adiposity, a phenotype termed “metabolically healthy obesity” (MHO) [4]. This population maintains favorable glucose and lipid metabolism, blood pressure, and inflammatory profiles compared to their counterparts with “metabolically unhealthy obesity” (MUO) [5,6]. Recent comprehensive reviews have further refined our understanding, with evidence suggesting that individuals with MHO still face moderately elevated cardiovascular risk compared to metabolically healthy normal-weight individuals [6].

While the identification of the MHO phenotype has challenged the paradigm that excess adiposity invariably leads to metabolic dysfunction, longitudinal evidence indicates that MHO is not completely benign and that a substantial proportion of individuals transition to metabolically unhealthy states over time [6,7,8,9,10]. To facilitate comparisons across phenotypes, we use metabolically healthy lean (MHL) synonymously with metabolically healthy normal weight (MHNW) throughout this manuscript. Where prior studies prefer MHNW, we retain the original term but note the equivalence to MHL.

Recent perspectives from leading researchers have questioned the term “healthy” in MHO. At the 2023 European Association for the Study of Diabetes meeting, Professor Matthias Blüher emphasized that individuals with MHO still have a 50% increased risk of coronary heart disease compared to metabolically healthy normal-weight individuals, suggesting that the term may be somewhat misleading [9]. This aligns with mounting evidence that MHO represents an intermediate state in disease progression rather than a stable protective phenotype. The comprehensive 2025 Nature Reviews Endocrinology review further reinforces this perspective, emphasizing that MHO should not be considered completely benign [6]. A landmark UK Biobank study of 381,363 participants with 11.2 years follow-up demonstrated that MHO participants had significantly higher rates of incident diabetes (HR 4.32), atherosclerotic CVD (HR 1.18), heart failure (HR 1.76), and all-cause mortality (HR 1.22) compared to metabolically healthy normal-weight individuals [10].

This comprehensive review examines the current knowledge surrounding MHO, including its various definitions, prevalence, clinical characteristics, underlying mechanisms, prognostic implications, and therapeutic considerations. By synthesizing evidence from diverse populations worldwide, we aim to provide a global perspective on this important phenotype while highlighting knowledge gaps and future research directions.

## 2. Definitions and Diagnostic Criteria

### 2.1. Traditional Criteria of MHO

Despite considerable research interest, there is no universally accepted definition of MHO, creating challenges for cross-study comparisons and clinical application. Most commonly, MHO is defined by the presence of obesity (typically BMI ≥ 30 kg/m^2^ in Western populations or ≥25 kg/m^2^ in Asian populations) coupled with the absence or limited presence of metabolic abnormalities [5].

Several criteria have been employed to define metabolic health in the context of obesity (Table 1). These include the following:

- The absence of metabolic syndrome components according to various definitions (e.g., NCEP ATP III, and IDF);
- Homeostasis model assessment of insulin resistance (HOMA-IR) below specific thresholds;
- Cardiometabolic risk factor clustering (including blood pressure, lipids, and glucose parameters);
- The absence of specific complications such as hypertension, dysglycemia, or dyslipidemia.

Recently, standardized criteria for MHO have been proposed that include the absence of cardiometabolic disease, favorable cardiometabolic blood profiles, normal blood pressure, absence of metabolic dysfunction-associated steatotic liver disease (MASLD), and absence of insulin resistance [6]. This definition attempts to consolidate previous approaches while incorporating emerging evidence on the importance of ectopic fat deposition, particularly in the liver, as a key determinant of metabolic health in obesity [6].

Similarly, “metabolically healthy normal-weight” (MHNW) individuals are typically defined as having a normal BMI (18.5–24.9 kg/m^2^) with no or minimal metabolic abnormalities. This group generally serves as the reference standard against which individuals with MHO are compared.

The heterogeneity in MHO definitions has substantial implications for research interpretation and clinical application. Different criteria yield varying prevalence estimates and potentially different associations with health outcomes. This definitional inconsistency remains a significant challenge in the field and highlights the need for harmonized criteria to facilitate more robust and comparable research.

Clinical implications of criterion heterogeneity: As summarized in Table 1, the choice of thresholds for glycemia, blood pressure, and lipids varies across definitions and materially alters MHO prevalence and risk estimates. For glycemia, some criteria require strictly normal fasting plasma glucose (FPG < 100 mg/dL) and HbA1c (<5.7%), whereas others tolerate one abnormality or use broader insulin-resistance indices (e.g., HOMA-IR) [5,6,11]. For blood pressure, cutoffs range from <130/85 mmHg to <140/90 mmHg and differ regarding antihypertensive treatment allowance: definitions permitting treatment generally classify fewer individuals as MHO [11,12]. For lipids, criteria vary by whether total cholesterol, LDL-C, HDL-C (sex-specific), and/or triglycerides are enforced and whether lipid-lowering therapy is allowed; stricter HDL-C and triglyceride thresholds (and disallowing therapy) reduce MHO prevalence and better discriminate cardiovascular risk [5,12,13]. Collectively, these differences explain much of the between-study heterogeneity and reinforce the need for harmonized, clinically practical criteria that extend beyond BMI to capture central adiposity and ectopic fat burden (e.g., waist–hip ratio in Zembic et al. [14] and liver fat in Section 5.5) [6,12,14].

**Table 1:** Comparison of various metabolically healthy obesity definitions and their diagnostic criteria.

Criterion	Meigs (2006) [15]	Karelis (2004/2008) [4,16]	Wildman (2008) [17]	Aguilar-Salinas (2008) [18]	Lynch (2009) [19]	BioSHaRE-EU (2014) Less Strict [20]	BioSHaRE-EU (2014) Strict [20]	Zembic et al. (2021) [14]	Wang et al. (2023) [21]
Insulin sensitivity/resistance									
HOMA-IR	≤2.5	≤1.95	≤5.13	-	-	-	-	-	-
Matsuda index	-	≥7.2	-	-	-	-	-	-	-
Blood pressure (mmHg)									
Systolic	<130	<140	<130	<140	<130	<140	<130	<130	<140
Diastolic	<85	<90	<85	<90	<85	<90	<85	-	<90
Anti-hypertensive medication	No	No	No	No	No	No	No	No	No
Glucose metabolism									
Fasting glucose (mg/dL)	<100	<100	<100	<100	<100	<110	<100	-	<100
2h glucose (mg/dL)	<140	-	-	-	-	-	-	-	-
HbA1c (%)	<5.7	-	-	-	-	-	-	-	-
Diabetes diagnosis	No	No	No	No	No	No	No	No	No
Glucose-lowering medication	No	No	No	No	No	No	No	-	No
Lipids									
Total or LDL cholesterol (mg/dL)	Total < 200	Total < 200	LDL < 130	Total < 200	Total < 200	-	-	-	Total < 240
HDL cholesterol (mg/dL)	>40 M, >50 F	>50	>40 M, >50 F	>40	>40 M, >50 F	>40 M, >50 F	>60 M, >70 F	-	>40 M, >50 F
Triglycerides (mg/dL)	<150	<150	<150	<150	<150	<150	<100	-	<150
Lipid-lowering medication	No	No	No	No	No	No	No	-	No
Anthropometric measures									
Waist circumference (cm)	<102 M, <88 F	-	<102 M, <88 F	-	<102 M, <88 F	<102 M, <88 F	<94 M, <80 F	-	<90 M, <85 F
Waist-to-hip ratio	-	-	-	-	-	-	-	<0.95 F, <1.03 M	-
Other biomarkers/indices									
C-reactive protein (mg/L)	-	-	<3.0	-	-	-	-	-	-
Number of criteria required	All	≥4	≤1	All	All	≤2	All	All	All

M = male; F = female; HOMA-IR = homeostasis model assessment of insulin resistance; NAFLD = non-alcoholic fatty liver disease. This table presents a comprehensive comparison of metabolically healthy obesity definitions used in major research studies from 2006 to 2023. The criteria encompass insulin sensitivity measures, blood pressure parameters, glucose metabolism markers, lipid profiles, anthropometric measurements, and inflammatory biomarkers. Notable variations include different HOMA-IR thresholds (ranging from ≤1.95 to ≤5.13), blood pressure cutoffs, and the number of criteria required for MHO classification. A definition requires meeting all criteria, while others (e.g., Wildman 2008 [17]) permit having one metabolic abnormality. The evolution of these definitions reflects the growing understanding of MHO complexity and the need for standardized diagnostic criteria.

### 2.2. Metabolic Dysfunction-Associated Steatotic Liver Disease as an Emerging Criterion in MHO Definition

Recent evidence increasingly suggests that metabolic dysfunction-associated steatotic liver disease (MASLD), formerly known as non-alcoholic fatty liver disease (NAFLD), should be considered a key component in defining metabolically healthy obesity. While traditional MHO definitions have not explicitly included liver fat assessment, emerging research highlights its critical importance in determining metabolic health trajectories.

A comprehensive review presented compelling evidence that MASLD should exclude an MHO diagnosis because hepatic steatosis represents an early predictor of metabolic dysfunction [22]. The review demonstrated that individuals with MHO and with MASLD show an increased risk of progression to metabolically unhealthy obesity and that BMI independently associates with progressive hepatic fibrosis regardless of metabolic phenotype [22]. This has led to recommendations for routine liver fat assessment in obesity classification.

Schulze and Stefan emphasize that liver fat content has been identified as one of the strongest predictors of the transition from MHO to MUO status [6]. Their review notes that “liver fat content has been identified as one of the strongest predictors of the transition from MHO to MUO” and suggests that assessing hepatic steatosis should become a standard component in the clinical evaluation of individuals with obesity, alongside traditional metabolic parameters [6].

Supporting this perspective, the Dallas Heart Study demonstrated that despite comparable BMI, individuals with MHO had approximately 60% lower hepatic triglyceride content than MUO subjects [23]. This reduced hepatic steatosis correlates with preserved hepatic insulin sensitivity and more favorable glucose and lipid metabolisms [23]. Similarly, in a 2015 overfeeding study, participants with MHO remained protected from hepatic insulin resistance despite weight gain, while participants with MUO showed a deterioration in liver metabolic function [24].

Recent advances in non-invasive biomarker development have facilitated MASLD assessment. A 2024 study identified circulating miR-122-5p, miR-151a-3p, miR-126-5p, and miR-21-5p as powerful biomarkers for early MASLD detection, showing significant associations with steatosis severity and liver stiffness [25]. Additionally, novel biomarker panels specifically targeting hepatic fibrosis detection demonstrate superior diagnostic performance compared to existing approaches [26].

This emerging understanding of hepatic steatosis’s central role in metabolic health may explain why some individuals maintain metabolic health despite obesity while others develop complications. In a meta-analysis of prospective cohort studies, MHO was related to an increased risk of type 2 diabetes compared with MHNW, even when the absence of hepatic steatosis was required in the definition, though this risk was substantially lower than in MHO with steatotic liver disease [27].

The practical implementation of liver fat assessment in routine clinical evaluation presents challenges, as advanced imaging techniques such as magnetic resonance spectroscopy or multiparametric MRI are not widely available. However, non-invasive biomarkers and scoring systems such as the Fatty Liver Index and Hepatic Steatosis Index, alongside transient elastography, may offer practical approaches for incorporating liver health assessment into standard MHO classification. Recent developments include point-of-care technologies like the NIS2+™ Test employing miR-34a-5p and YKL-40 for comprehensive MASLD monitoring [28].

As our understanding of metabolically healthy obesity continues to evolve, future standardized definitions will likely incorporate hepatic steatosis assessment, representing an important advancement in risk stratification and personalized treatment approaches for individuals with obesity.

## 3. Global Prevalence and Demographic Characteristics

The estimated prevalence of MHO varies considerably depending on the definition employed, the population studied, and demographic factors. Using commonly applied criteria, global data suggest that approximately 10–30% of individuals with obesity may be classified as metabolically healthy [29]. Recent meta-analyses confirm that approximately 35% of individuals with obesity are metabolically healthy, though 49% develop metabolic abnormalities within 10 years [30].

A meta-analysis comprising 40 population-based studies from around the world found that MHO prevalence ranged from 6% to 75%, highlighting the substantial influence of definitional criteria. When applying more stringent definitions requiring the absence of any metabolic abnormalities, prevalence estimates trend toward the lower end of this range.

Several demographic factors influence MHO prevalence:

- Sex: Most studies report a higher MHO prevalence among women compared to men, potentially reflecting sex differences in body fat distribution and adipose tissue function [6]. Recent discoveries highlight significant sex-specific mechanisms, including the identification of CTRP10’s female-specific role in maintaining metabolic health during obesity [31]. A 2024 study demonstrated that adipose tissue insulin resistance is more pronounced in men than women, with men showing 10-fold lower insulin sensitivity and decreased adipose expression of insulin receptor substrate 1 (IRS1) [32]. Analysis of 9631 Saudi adults found females had significantly higher age-adjusted prevalence of MHO than males (OR = 1.22, 95% CI 1.1–1.4, *p* = 0.009) [33]. These sex-specific differences extend to therapeutic responses, with emerging evidence suggesting that women and men may respond differently to interventions aimed at maintaining or restoring metabolic health in obesity.
- Age: MHO is more common in younger individuals and decreases with advancing age, suggesting that metabolic health may deteriorate over time despite stable weight [6]. A longitudinal study of 9809 individuals found that genetic predisposition to higher BMI may protect against MHO conversion, though lifestyle factors did not predict conversion over 4 years [8].
- Ethnicity: Significant ethnic differences exist in MHO prevalence. Studies from Europe and North America have documented higher rates in populations of European descent compared to those of African, Hispanic, or South Asian ancestry [6]. A study examining 2350 Asian individuals with a BMI ≥ 25 kg/m^2^ reported that 13.3% met MHO criteria [34]. Recent analysis of Arab populations revealed leptin resistance as central to MHO-to-MUO progression, with significantly higher leptin levels in metabolically unhealthy groups [35].
- BMI: The prevalence of MHO typically decreases with increasing severity of obesity. Grade 1 obesity (BMI 30–34.9 kg/m^2^) shows approximately twice the prevalence of MHO compared to morbid obesity (BMI ≥ 40 kg/m^2^) [6].

These demographic patterns suggest that genetic, hormonal, adipose tissue distribution, and environmental factors likely interact to determine metabolic health in obesity. Understanding these differences may provide insights into the underlying mechanisms of metabolic protection or susceptibility.

## 4. Clinical Characteristics and Health Outcomes

Despite being termed “metabolically healthy,” considerable evidence indicates that individuals with MHO still face elevated health risks compared to metabolically healthy normal-weight counterparts. This section examines the clinical characteristics and long-term health outcomes associated with MHO.

### 4.1. Type 2 Diabetes Risk

Multiple prospective studies and meta-analyses have demonstrated that individuals with MHO have a significantly increased risk of developing type 2 diabetes compared to MHNW individuals, though this risk is lower than for those with MUO. While MUO is associated with a 5–20-fold increased risk of developing type 2 diabetes compared to MHNW, MHO still carries a 4-fold higher risk than MHNW [36].

A meta-analysis reported that MHO had a relative risk of 3.00 (95% CI: 2.33–3.85) for incident type 2 diabetes compared to MHNW individuals [37]. This elevated risk persisted even in studies with longer follow-up periods, suggesting that MHO does not represent complete protection against diabetes development [37]. Notably, even individuals with MHO and with steatotic liver disease showed increased risk compared to MHNW subjects [37].

Recent evidence provides deeper molecular insights into these differences. A comprehensive 2024 study evaluating over 100 cardiometabolic outcomes found that MHO is characterized by reduced ceramide content in skeletal muscle, increased expression of genes involved in branched-chain amino acid catabolism and mitochondrial structure/function, decreased expression of genes involved in inflammation and extracellular matrix remodeling in adipose tissue, and increased expression of genes involved in adipogenesis compared to counterparts with MUO [38].

### 4.2. Cardiovascular Disease Risk

The relationship between MHO and cardiovascular outcomes has been extensively investigated, with somewhat mixed results, though most evidence points to increased risk [7,11,39,40,41]. A recent meta-analysis found that participants with MHO had increased cardiovascular event risk compared to healthy normal-weight participants (pooled RR 1.45, 95% CI 1.20–1.70), with risk particularly elevated during long-term follow-up [13].

Advanced cardiovascular imaging studies have revealed that individuals with MHO display subclinical cardiac abnormalities. MRI analysis shows that myocardial fibrosis is greatest in MUO, followed by MHO, then MHL (metabolically healthy lean; synonymous with MHNW), with MHO showing potential subclinical contractile and diastolic dysfunction [42].

The UK Biobank study, analyzing data from 381,363 participants, demonstrated that individuals with MHO had significantly higher risks of heart failure (HR 1.76), diabetes (HR 4.32), and all-cause mortality (HR 1.22) compared even to metabolically unhealthy normal-weight individuals [10]. A 2025 meta-analysis also found that metabolically healthy obesity associates with greater atrial fibrillation risk than normal weight but lower risk than metabolically unhealthy obesity [43].

Recent evidence suggests that the definition of MHO significantly impacts cardiovascular risk assessment. When using common definitions, MHO shows moderately increased cardiovascular risk compared to MHNW, but when incorporating waist–hip ratio (a measure of central adiposity) into the definition, individuals meeting MHO criteria show substantially lower cardiovascular risk [12].

Research in Japanese populations indicates that MHO alone does not significantly increase cardiovascular disease risk, but when combined with abdominal obesity, risks of myocardial infarction, angina, heart failure, and atrial fibrillation increase [44].

Some researchers have expressed concern about the term “healthy” in MHO, as excessive fat accumulation may adversely affect the cardiovascular system independently of typical metabolic profile abnormalities [9,10].

### 4.3. Chronic Kidney Disease Risk

The association between MHO and renal outcomes has gained increasing attention. A large Korean cohort study of 62,249 metabolically healthy individuals demonstrated that chronic kidney disease risk increased progressively across BMI categories, with MHO showing intermediate risk between MHNW and MUO [45].

Another 20-year follow-up of 8731 Korean individuals found that MHO had a significantly higher risk of developing chronic kidney disease than MHL [46]. Meta-analyses confirm that MHO is associated with increased chronic kidney disease risk (relative risk 1.48, 95% CI: 1.32–1.66) compared to MHNW individuals [47].

### 4.4. Cancer Risk

Obesity-induced hyperinsulinemia and chronic inflammation may promote cancer development and proliferation. Obesity has been associated with increased risks of esophageal, thyroid, liver, pancreatic, and colorectal cancers.

Analysis of 23,630 individuals from Europe and the United States found that MHO was associated with an increased risk for several obesity-related cancers [48]. A meta-analysis reported that MHO was associated with a 17% increased overall cancer risk compared to MHNW (relative risk 1.17, 95% CI: 1.01–1.35) [49].

These findings suggest that excess adiposity may promote carcinogenesis through mechanisms partially independent of metabolic dysfunction, potentially including altered sex hormone metabolism, chronic inflammation, and adipokine dysregulation.

## 5. Pathophysiological Mechanisms Underlying MHO

**Figure 1:**
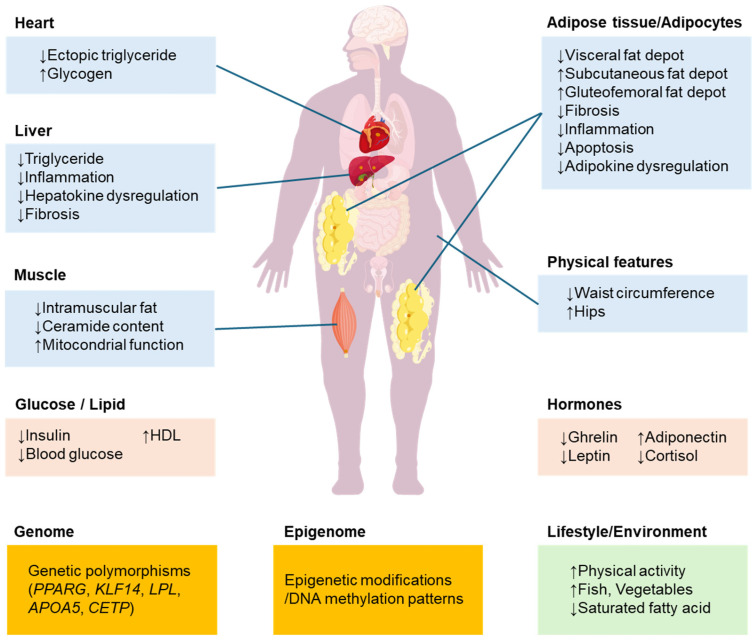
Updated overview of pathophysiological features characterizing metabolically healthy obesity. This figure has been redrawn to remove blank space and improve clarity. Up arrow: increase, Down arrow: decrease.

The biological mechanisms underlying metabolic health despite obesity remain incompletely understood but appear to involve multiple interacting factors. Current evidence points to several key mechanisms that may contribute to the MHO phenotype (Figure 1).

### 5.1. Adipose Tissue Distribution Patterns

Body fat distribution, particularly the relative proportion of subcutaneous versus visceral adipose tissue, plays a critical role in determining metabolic health. Under physiological conditions, white adipose tissue expansion occurs through both hypertrophy (increased size) of existing adipocytes and increased adipocyte numbers. The expansion capacity of subcutaneous white adipose tissue is suggested as a major determinant of obesity-related metabolic abnormalities [50].

When white adipose tissue expansion is impaired, ectopic fat accumulation in non-adipose tissues is promoted, contributing to the development of insulin resistance. For example, hepatic steatosis is greater in MUO than MHO [51], and liver fat correlates with insulin resistance independently of BMI, body fat percentage, and visceral fat content, consistent with hepatic steatosis formation inhibiting MHO development [51].

When matched for BMI and sex, although body fat percentage does not differ between MHO and MUO, MHO has less visceral fat than MUO [52]. Reports also indicate that women with MHO have more subcutaneous fat in the thigh or lower leg compared to women with MUO [53].

Genetic determinants of fat distribution have been analyzed, with data supporting that sufficient expansion capability of gluteal-femoral adipose tissue is a determinant of MHO [54,55]. Another report identified *SNZ10* as the gene most strongly associated with waist–hip ratio adjusted for BMI in women [56]. A 2024 genome-wide association study of 93,673 Korean subjects revealed distinct genetic components distinguishing MHO from metabolically unhealthy obesity, with heterogeneous patterns on chromosomes 3 and 11 [57].

### 5.2. Adipose Tissue and Adipocyte Function

Beyond distribution patterns, the functional characteristics of adipose tissue appear to differ substantially between individuals with MHO and MUO. A study provided a comprehensive cellular mapping of subcutaneous and visceral adipose tissue from individuals with MHO and MUO. This single-nucleus RNA-sequencing analysis, combined with spatial transcriptomics, revealed that “mesothelial cells, adipocytes, and adipocyte-progenitor cells exhibit the strongest correlation with metabolic disease” [58].

The study demonstrated that visceral adipose tissue plasticity is key to maintaining metabolic health despite excess adiposity [58]. Specifically, mesothelial-to-mesenchymal transition uncouples obesity from metabolic dysfunction, providing cellular targets for therapeutic intervention [58]. This represents a paradigm shift from viewing MHO as simply “less inflamed” fat to understanding it as having a fundamentally different cellular architecture [58].

Additional single-cell RNA sequencing studies have identified four distinct adipose progenitor cell subsets in human visceral adipose tissue, with CD9+CD55low APCs significantly increased in T2D patients and positively correlated with fasting glucose and HbA1c [59]. These pathogenic cells secrete PEDF (pigment epithelium-derived factor), creating a detrimental niche promoting adipocyte lipolysis and glucose homeostasis impairment. Furthermore, a unique population of mesothelial-like cells in human omental adipose tissue has been shown to hinder adipogenesis through high IGFBP2 expression [60].

Importantly, when comparing metabolically healthy non-obese individuals to those with MHO, no major cellular compositional differences were found in visceral or subcutaneous adipose tissue, suggesting that “healthy adipose tissue expansion is strongly linked to preserved systemic nutrient homeostasis despite obesity” [58]. This observation is particularly striking as it indicates that adipose tissue dysfunction, rather than adiposity itself, is the critical determinant of metabolic health.

Distinct cell populations and transcriptional programs in adipose tissue have been identified that are strongly linked to metabolic disease [58]. Visceral adipose tissue undergoes significant remodeling in different health states, while subcutaneous adipose tissue shows more subtle changes. The most prominent differences were evident in mesothelial cells, which were over-represented in the visceral adipose tissue of individuals with MHO, a finding that had not been previously reported in the context of metabolic health [58].

#### 5.2.1. Lipogenic Capacity

Subcutaneous fat in MHO shows higher gene expression of molecules promoting lipogenesis (*CD36*, *GLUT4*, *CHREBP*, *FASN*, and *MOGAT1*) compared to MUO, with expression levels correlating with insulin sensitivity [5]. This enhanced capacity for triglyceride synthesis and storage may protect against ectopic lipid deposition in the liver, muscle, and other tissues.

Compared to MUO, MHO shows reduced ceramide content in skeletal muscle, increased expression of genes involved in branched-chain amino acid catabolism and mitochondrial structure/function, decreased expression of genes involved in inflammation and extracellular matrix remodeling in adipose tissue, and increased expression of genes involved in adipogenesis [38].

#### 5.2.2. Adipose Tissue Inflammation

In MHO, adipose tissue exhibits a distinct immunometabolic profile that limits systemic inflammation and cardiovascular risk. Specifically, reduced infiltration of pro-inflammatory macrophages and lower secretion of adipocytokines such as TNF-α and IL-6 contribute to a more favorable inflammatory milieu. Additionally, elevated levels of anti-inflammatory adipokines like adiponectin help maintain vascular integrity and metabolic homeostasis, explaining the reduced incidence of CVD in MHO despite excess adiposity.

In obesity, inflammatory cells such as macrophages infiltrate adipose tissue, inducing chronic inflammation and insulin resistance. MHO shows milder macrophage infiltration in both subcutaneous and visceral fat compared to MUO, with a lower proportion of pro-inflammatory macrophages [61].

Blood concentrations of inflammatory cytokines (PAI-1, IL-6, and TNF-α) are also reported to be lower in MHO than MUO. A 2025 cross-sectional study of 450 Saudi adults revealed that while individuals with MHO showed lower inflammatory markers compared to metabolically unhealthy obesity, they still demonstrated subclinical inflammation and adipose dysfunction compared to metabolically healthy normal weight [35]. In MHO, the proportion of inflammatory lymphocytes Th17 and Th22 cells among CD4-positive T cells is lower than in MUO [62,63], and anti-inflammatory CD4-positive Th2 cell numbers correlate with insulin sensitivity [64].

#### 5.2.3. Healthy Versus Unhealthy Adipose Expansion

Recent studies underscore the dual nature of WAT remodeling in obesity. In metabolically healthy obesity (MHO), adaptive remodeling of white adipose tissue (WAT) enhances angiogenesis, preserves mitochondrial integrity, and supports healthy adipocyte expansion, thereby reducing ectopic fat deposition and insulin resistance. Conversely, in metabolically unhealthy obesity (MUO), maladaptive WAT remodeling—characterized by fibrosis, impaired vascularization, and mitochondrial dysfunction—promotes ectopic lipid accumulation and systemic insulin resistance. These findings highlight the pivotal importance of intervening in WAT remodeling to preserve metabolic health and prevent the transition from MHO to MUO.

Recent research has further clarified the mechanistic distinction between healthy and unhealthy adipose tissue expansion. It has been explained that “expansion of healthy adipose tissue is accompanied by adequate capillary angiogenesis and mitochondria-centered metabolic integrity, whereas expansion of unhealthy adipose tissue is associated with capillary and mitochondrial derangement” [65]. This vascular and mitochondrial health appears critical to maintaining metabolic homeostasis despite obesity [65].

In healthy adipose expansion, mitochondrial content in growing adipose tissue is preserved, supporting proper energy homeostasis through the regulation of lipid turnover, adipogenesis, adipokine secretion, and metabolic substrate utilization [65]. Adequate production of mitochondrial reactive oxygen species is beneficial for insulin sensitivity, adipocyte differentiation, and adipose tissue function, while dysregulated mitochondrial function contributes to metabolic dysfunction [65].

Exercise is highlighted as “a potent behavioral intervention for preventing and reducing obesity and other metabolic diseases” [65]. Exercise appears to impose unique physiological stimuli that can alter angiogenesis and mitochondrial remodeling in adipose tissues, potentially promoting healthy adipogenesis [65]. Studies in mice using 2H labeling techniques suggest that exercise may inhibit the generation of new adipocytes and extend the lifespan of existing adipocytes, potentially contributing to MHO [66]. A 2024 systematic review found that concurrent training combining aerobic and resistance exercise was most effective in reducing BMI, body fat composition, and C-reactive protein in individuals with MHO [67].

#### 5.2.4. Adipocyte-Specific Gene-Modified Animals

MHO has been observed in adipocyte-specific gene-modified animals. Adipocytes produce various collagens that inhibit adipose tissue expansion. One mechanism of this inhibition is physical restriction of adipocyte expansion by collagen proliferation in adipose tissue [68]. *Col6* (collagen VI)-deficient mice show enhanced adipocyte expansion under high-fat diet conditions and, despite increased adipose tissue weight compared to control mice, maintain insulin sensitivity and suppress adipose tissue inflammation. This process represents a form of “healthy adipose expansion,” where adipose tissue expands without exacerbation of inflammation or fibrosis, leading to increased capacity for lipid accumulation and preventing ectopic fat deposition in other organs.

Similarly, adipocyte-specific glucocorticoid receptor knockout (AGRKO) mice exhibit MHO phenotypes when challenged with excess glucocorticoids. These mice display healthy adipose expansion with diminished ectopic lipid deposition and improved insulin sensitivity [69]. Additionally, adipocyte-specific PTEN knockout mice show enhanced insulin signaling in adipose tissue, which promotes beneficial adipose expansion, reduces hepatic fat accumulation, and improves systemic metabolic homeostasis despite increased weight gain [70]. Additional transgenic models exhibiting MHO phenotypes include mice with adipose tissue-specific overexpression of *Slc2a4* (GLUT4) [71], *Adipoq* (adiponectin) [72], mitoNEET [73], and *Tnmd* (tenomodulin) [74]. These various models generally show smaller adipocytes, increased cell numbers, reduced adipose tissue inflammation, suppressed ectopic fat accumulation, and maintained insulin sensitivity.

However, many of these gene-modified mice use the *Adipoq* promoter, inducing specific gene expression regulation primarily in mature adipocytes. Therefore, it remains challenging to fully distinguish whether the phenotype results from changes in the function of individual adipocytes themselves or from secondary changes due to increased adipocyte numbers. Nevertheless, these findings suggest that adipose tissue/cell function is more important than fat accumulation itself as a determinant of metabolic abnormalities.

### 5.3. Genetic and Epigenetic Factors

#### 5.3.1. Key Single-Nucleotide Polymorphisms

While numerous animal experiments report models exhibiting MHO phenotypes through single gene modifications, no consistent gene mutations or polymorphisms correlating with MHO have been identified in humans. Leptin gene (G19A and A19A) and adiponectin gene (G276G, G276T, etc.) genotypes do not differ between MHO and MUO [75].

Recent research has identified promising genetic markers that may differentiate MHO from MUO phenotypes. A 2023 study of Hungarian adults identified 19 SNPs (in 15 genes) whose combined effect was strongly associated with increased risk of MUO (OR = 1.77, *p* < 0.001) [76]. Four SNPs in particular (rs10838687 in *MADD*, rs693 in *APOB*, rs1111875 in *HHEX*, and rs2000813 in *LIPG*) significantly increased MUO risk (OR = 1.76). These genes clustered into three functional groups: genes primarily related to lipid metabolism (*ADIPOQ*, *APOB*, *CETP*, *LIPC*, *LIPG*, and *LPL*), adipocyte development (PPARG), and glucose metabolism/diabetes risk (*C2CD4B*, *CDKN2B*, *GIPR*, *HHEX*, *SLC2A2*, and *SLC30A8*) [76].

Recent integrated genetic and epigenetic analyses have uncovered *GLP1R* (glucagon-like peptide-1 receptor) associations with the MHO phenotype, providing insights into therapeutic targets [77]. KLF14 has been identified as another important genetic determinant of MHO. KLF14 is an imprinted gene encoding a transcription factor discovered to regulate adipose tissue expression. Human carriers of the KLF14 type 2 diabetes risk allele show a shift in body fat from gluteal–femoral to abdominal regions, with this genetically determined change in fat distribution observed only in women [78].

#### 5.3.2. Population-Specific Genetic Associations

Genetic associations with MHO show notable population-specific patterns: *KCNQ1* variants in Chinese children [79], *FTO* polymorphisms in Iranians [80], *MC4R* polymorphisms in Chinese populations [81], and *PPARG* gene polymorphisms in Chinese children [79]. A genome-wide association study of nearly 50,000 Koreans found polymorphisms in the *LPL*, *APOA5*, and *CETP* genes associated with higher risk of metabolically unhealthy phenotypes [82].

#### 5.3.3. Epigenetic Contributions

Emerging evidence suggests that epigenetic modifications may also play a crucial role in determining metabolic health in obesity. A prospective study identified 26 CpG sites significantly differentially methylated between stable and unstable MHO, with higher methylation at cg20707527 (*ZFPM2*) showing protective effects against progression, while higher methylation at cg11445109 (*CYP2E1*) increased progression risk to metabolically unhealthy obesity [83]. A 2022 European study highlighted the importance of DNA methylation patterns in differentiating MHO from MUO phenotypes, suggesting that epigenetic markers could serve as potential biomarkers for metabolic health independent of BMI [84]. Additionally, recent work has identified specific epigenetic signatures associated with the maintenance of metabolic health despite excess adiposity, further supporting the complex interplay between genetic predisposition and environmental factors in determining MHO status [77].

#### 5.3.4. Gene–Lifestyle Interactions

A significant 2024 study provides compelling evidence that genetic predisposition interacts with lifestyle factors to determine obesity risk [85]. This large-scale analysis of 338,645 UK Biobank participants demonstrated that high genetic risk and obesogenic lifestyle jointly raise obesity risk with significant additive interaction (*p* for interaction < 0.001). The study found that the absolute differences in obesity risk between those with healthy versus poor lifestyles expanded as genetic risk increased, suggesting that lifestyle modification is particularly important for genetically predisposed individuals [85].

Most importantly for clinical application, this growing body of evidence suggests that genetic risk is not deterministic. High genetic predisposition to obesity increases risk of obesity-related morbidities, but healthy lifestyle can prevent these morbidities even in individuals with high genetic risk. The UK Biobank study demonstrated that individuals with high genetic risk but healthy lifestyle experienced comparable risks of obesity-related morbidities to individuals without high genetic risk, whereas those with high genetic risk and poor lifestyle showed significantly increased disease burden [85].

These findings collectively suggest that genetic variants and epigenetic modifications affecting lipid metabolism, fat distribution, adipogenesis, and glucose homeostasis contribute to determining metabolic health in obesity. While genetic factors increase susceptibility, lifestyle modifications appear capable of substantially offsetting genetic risk. This interaction between genes, epigenetics, and environment underscores the potential value of polygenic risk scoring in identifying high-risk individuals who might benefit most from targeted lifestyle interventions.

#### 5.3.5. Epigenetic Memory of Obesity

A recent landmark study demonstrated that adipose tissues retain cellular transcriptional changes after significant weight loss, revealing persistent obesity-induced epigenome alterations in adipocytes that negatively affect function and metabolic response [86]. Mice carrying this “obesogenic memory” showed accelerated weight regain, providing molecular evidence for why MHO often represents a transient state.

### 5.4. Skeletal Muscle Characteristics

Skeletal muscle metabolism plays a crucial role in glucose homeostasis and lipid handling. individuals with MHO demonstrate several favorable muscle characteristics:

- Enhanced Mitochondrial Function: Muscle biopsies from subjects with MHO show higher mitochondrial content, enhanced oxidative enzyme activity, and more efficient respiratory chain function compared to individuals with MUO [38]. These differences correlate with improved insulin sensitivity and reduced intramyocellular lipid accumulation.
- Reduced Ectopic Lipid Deposition: Despite obesity, individuals with MHO maintain lower intramyocellular lipid content and altered lipid composition, with reduced ceramide and diacylglycerol species known to interfere with insulin signaling [38].
- Preserved Insulin Signaling: Molecular analyses of skeletal muscle from individuals with MHO demonstrate preserved insulin receptor substrate (IRS) phosphorylation and downstream Akt/PKB activation compared to subjects with MUO [52].

### 5.5. Liver Fat Content and Function

Metabolic dysfunction-associated steatotic liver disease (MASLD) represents a critical determinant of metabolic health in obesity. Multiple studies have demonstrated that individuals with MHO have significantly lower hepatic triglyceride content than BMI-matched subjects with MUO [23,51].

The Dallas Heart Study, using magnetic resonance spectroscopy to quantify hepatic triglyceride content, found that despite comparable BMI, individuals with MHO had approximately 60% lower liver fat content than subjects with MUO [23]. This reduced hepatic steatosis correlates with preserved hepatic insulin sensitivity and more favorable glucose and lipid metabolism.

Liver fat content has been identified as one of the strongest predictors of the transition from MHO to MUO [6]. Assessing hepatic steatosis should be a standard component in the clinical evaluation of individuals with obesity, alongside traditional metabolic parameters. Cross-sectional studies demonstrate increased prevalence of hepatic steatosis and fibrosis in subjects with MHO compared to metabolically healthy non-obese individuals, with longitudinal data showing that increasing BMI independently associates with MASLD incidence and progressive hepatic fibrosis [22].

Beyond simple fat content, liver inflammation and fibrosis also appear reduced in MHO compared to MUO, as evidenced by lower circulating hepatic enzymes (ALT, AST, and GGT) and reduced fibrosis markers [87].

Circulating fibroblast growth factor 21 (FGF21), primarily released from the liver, is a key biomarker for differentiating MHO from MUO. In individuals with MUO, elevated FGF21 levels indicate a state of FGF21 resistance, where impaired signaling contributes to adipose dysfunction and insulin resistance. Conversely, the lower levels in MHO are characteristic of preserved FGF21 signaling [88]. Recent metabolomic investigations have identified consensus biomarker groups including essential amino acids, energy metabolites, gut microbiota metabolites, acylcarnitines, and lysophosphatidylcholines for MASLD assessment [89].

### 5.6. Lifestyle Factors

#### Nutritional Status and Targeted Dietary Interventions in MHO

Nutritional status influences whether excess adiposity progresses to metabolic dysfunction. Dietary lipid composition appears particularly relevant for adipose tissue expandability and inflammatory tone. In metabolically healthy obesity, distinct fatty-acid profiles correlate with a lower inflammatory state [90], and individuals with MHO often report higher intakes of fish and vegetables with lower saturated fat and sugar-sweetened beverages versus MUO [91,92]. Mechanistically, remodeling of adipocyte membrane phospholipids through the PPARγ–LPCAT3 axis links dietary n-6 polyunsaturated fatty acids to improved adipose storage capacity and insulin sensitivity, offering a causal framework for diet quality shaping adipose function [93]. From a practical standpoint, dietary patterns emphasizing unprocessed foods, higher PUFAs (particularly marine sources), fiber-rich vegetables/legumes, and reduced refined sugars and saturated fats are associated with more favorable lipid profiles, lower ectopic fat, and attenuated inflammation in people with obesity, including those with MHO [90,91,92,93]. Importantly, these benefits often occur independent of large weight loss, complementing the protective effects of physical activity described above.

The suggested clinical approach for MHO maintenance and MUO prevention is as follows:

- Prioritize diet quality (Mediterranean-like pattern), with ≥2 fish meals/week, abundant non-starchy vegetables, legumes, nuts, and whole grains and limit SSBs and processed meats [90,91,92];
- Replace saturated fats with PUFA-rich options (e.g., canola/soy/sunflower oils and fatty fish) to support adipose tissue expandability and insulin sensitivity mechanistically via PPARγ–LPCAT3 [93];
- Combine diet quality improvements with regular aerobic and resistance exercise, which synergistically remodels adipose vasculature and mitochondria to favor healthy adipose expansion [65,66,67].

Together, these data suggest that targeted nutritional strategies are integral to preserving metabolic health in obesity and may delay or prevent transition from MHO to MUO [30,94].

Several lifestyle factors have been associated with the MHO phenotype, though their causal role remains incompletely established:

- Physical Activity: Individuals with MHO typically report higher levels of both leisure time and total physical activity compared to subjects with MUO [95]. A meta-analysis of 15 studies found that individuals with MHO engaged in approximately 30% more moderate-to-vigorous physical activity than their metabolically unhealthy counterparts. Meta-analysis of physical activity studies found that individuals MHO demonstrate higher physical activity levels, reduced sedentary behavior, and superior cardiorespiratory fitness compared to metabolically unhealthy obesity individuals, with higher fitness levels potentially preventing transition to metabolically unhealthy states [94]. Recent studies examining the effects of exercise on adipose tissue have revealed that “exercise is a potent behavioral intervention for preventing and reducing obesity and other metabolic diseases”: exercise appears to impose unique physiological stimuli that can alter angiogenesis and mitochondrial remodeling in adipose tissues, potentially promoting healthy adipogenesis. Studies in mice using 2H labeling techniques suggest that exercise may inhibit the generation of new adipocytes and extend the lifespan of existing adipocytes, potentially contributing to MHO [66].
- Cardiorespiratory Fitness: Independent of self-reported physical activity, measured cardiorespiratory fitness is significantly higher in individuals with MHO versus MUO. The HERITAGE Family Study demonstrated that higher baseline fitness and greater fitness improvements with exercise training predicted transition from MUO to MHO status.
- Dietary Patterns: Specific nutritional biomarkers (particularly carotenoids and vitamin D) are reportedly useful characteristics of MHO [90]. Some studies report that individuals with MHO consume more fish and vegetables and fewer sugar-sweetened beverages and saturated fatty acids [91], though others find no clear differences in total energy intake and nutrient intake between MHO and MUO [92]. Recent molecular insights have revealed how specific dietary lipid composition influences adipose tissue function. The PPARγ-LPCAT3 pathway demonstrates that dietary n-6 PUFA intake directly modulates adipose tissue expandability through membrane lipid remodeling [93]. This finding suggests that the quality of dietary fats, particularly the omega-6 PUFA content, may be as important as total fat intake in determining metabolic health outcomes in obesity. While this area requires further research in human populations, it provides a mechanistic basis for understanding how dietary composition influences the MHO phenotype.
- Sleep Quality: Some evidence suggests that individuals with MHO maintain more favorable sleep patterns, with lower prevalence of sleep disorders and more consistent sleep duration compared to subjects with MUO.

These lifestyle factors may contribute to MHO both directly and through interactions with genetic predisposition, highlighting the complex nature of metabolic health determinants in obesity.

## 6. Temporal Stability and Transition Patterns

A critical consideration regarding MHO is its temporal stability. Longitudinal studies consistently demonstrate that many individuals initially classified as MHO transition to MUO over time, raising questions about whether MHO represents a truly stable phenotype or merely a transient state.

### 6.1. Transition Rates and Patterns

Multiple prospective cohort studies have documented substantial transition rates from MHO to MUO over time. A longitudinal study of 6809 individuals from diverse ethnic backgrounds found that over a median follow-up of 12.2 years, more than 50% of initially individuals with MHO progressed to MUO [96].

A study of Chinese adults showed that over 30% of MHO transitioned to MUO within just 2 years [97]. The Nurses’ Health Study, tracking 90,257 women over 30 years, reported that 84% of initially women with MHO eventually transitioned to MUO [7]. Meta-analyses indicate that approximately 49% of individuals with MHO develop metabolic abnormalities within 10 years [30].

These observations have led many researchers to consider MHO as merely an intermediate state in the progression from weight gain to the development of insulin resistance and metabolic syndrome rather than a stable protective phenotype [6] [29].

The transient nature of MHO necessitates regular monitoring and proactive management strategies even in metabolically healthy individuals with obesity [6].

### 6.2. Predictors of Transition

Several baseline characteristics have been identified as predictors of transition from MHO to MUO:

- Age: Older individuals have significantly higher transition rates, with higher age associated with increased transition risk [97].
- BMI: Higher baseline BMI predicts greater likelihood of transitioning to MUO [97].
- Liver Fat Content: Baseline hepatic steatosis strongly predicts transition from MHO to MUO. Metabolic dysfunction-associated steatotic liver disease has been identified as an important factor in this transition, with liver fat content closely related to insulin resistance progression [22,97].
- Epigenetic Factors: Recent evidence shows that specific DNA methylation patterns predict transition, with 26 CpG sites differentially methylated between stable and unstable MHO [83].

### 6.3. Health Consequences of Transition

The health implications of transitioning from MHO to MUO appear substantial. A meta-analysis of eight cohort studies demonstrated that individuals who transitioned from MHO to MUO had a 42% higher risk of cardiovascular events compared to those who maintained stable MHO status (relative risk 1.42; 95% CI: 1.24–1.60) [98].

The Nurses’ Health Study found that even among those maintaining MHO status long-term, cardiovascular disease risk remained elevated compared to metabolically healthy lean participants [7].

These findings suggest that metabolic health surveillance and intervention strategies aimed at preventing transition from MHO to MUO may represent an important clinical approach for reducing obesity-associated complications.

## 7. Pharmacological and Non-Pharmacological Interventions

Given the increased disease risk associated with MHO compared to MHNW, alongside the high transition rate to MUO over time, the question of appropriate interventions for individuals with MHO has generated considerable debate and research.

### 7.1. Lifestyle Interventions

Beyond energy restriction, diet quality per se contributes to metabolic improvements in MHO. Patterns characterized by higher PUFAs and carotenoid-rich foods with reduced saturated fats and added sugars are associated with lower inflammation and more favorable adipose biology in MHO [90,91,92,93]. In meta-analytic findings and controlled studies, combining energy-restricted meal plans with structured exercise yields the most consistent improvements in triglycerides, HDL-C, glycemia, and inflammatory markers [30,67,92].

- Weight Loss: The impact of weight loss interventions in MHO remains somewhat controversial. Some studies suggest that weight reduction in individuals with MHO leads to improvements in inflammatory markers, liver fat content, and insulin sensitivity [99]. However, other studies have reported minimal metabolic benefits or even paradoxical worsening of certain parameters following weight loss in subjects with MHO [4].
- The HERITAGE Family Study found that individuals with MHO showed smaller improvements in insulin sensitivity and lipid profiles following weight loss compared to subjects with MUO, suggesting potentially different response patterns [92]. This has raised questions about the risk-benefit ratio of aggressive weight loss interventions in all individuals with MHO.
- Physical Activity: Exercise interventions appear particularly beneficial for individuals with MHO, often producing metabolic improvements independent of significant weight loss. A randomized controlled trial demonstrated that six months of moderate-intensity exercise in subjects with MHO led to significant reductions in visceral fat, liver fat, and systemic inflammation despite minimal changes in body weight [92]. A meta-analysis of seven intervention studies found that energy-restricted diet interventions combined with exercise effectively improved metabolic profiles for individuals with MHO [30].

Research shows that lifestyle interventions including both diet and physical activity can help prevent the transition from MHO to MUO. Studies indicate that approximately 30% to 50% of people with MHO convert to MUO after 4 to 20 years of follow-up, but physical activity may prevent the development of both MHO and MUO and helps increase the transition from MUO to MHO [100]. Interventions focusing on higher physical activity and lower fat intake can help prevent the transition to MUO [101], though the risk of transitioning from MHO to MUO is greater in those with older age, evidence of more severe metabolic dysfunction, a poor lifestyle index, and weight gain during the observation period.

### 7.2. Pharmacological Approaches

Several medication classes have demonstrated potential for maintaining or improving metabolic health in obesity:

- Thiazolidinediones: These PPARγ agonists promote subcutaneous adipocyte differentiation and reduce ectopic fat deposition. Clinical trials have shown that pioglitazone and rosiglitazone can induce an “MHO-like” phenotype, characterized by increased subcutaneous fat but reduced liver fat and improved insulin sensitivity [102,103].
- SGLT2 Inhibitors: Emerging evidence from both animal models and clinical studies suggests that sodium-glucose cotransporter-2 (SGLT2) inhibitors may promote “healthy adipose expansion” while reducing ectopic fat deposition and improving metabolic parameters [104,105,106]. In metabolic dysfunction-associated steatohepatitis models, SGLT2 inhibitors have been shown to attenuate hepatic steatosis, inflammation, and fibrosis despite minimal weight reduction or even adipose tissue expansion [105].
- GLP-1 Receptor Agonists and GIP/GLP-1 Dual Agonists: The pharmaceutical landscape for obesity treatment is experiencing an unprecedented transformation. These agents appear to induce favorable changes in body composition beyond simple weight reduction. Recent studies with tirzepatide, a GIP/GLP-1 receptor dual agonist, have demonstrated preferential reduction in visceral fat compared to subcutaneous fat, potentially promoting a more metabolically favorable fat distribution pattern [107]. A comprehensive 2024 review highlights tirzepatide achieving up to 22.5% weight loss, with emerging triple agonists like retatrutide showing even greater efficacy [108]. The dual GIP/GLP-1 agonist could reduce cardiovascular risk and prevent conversion to metabolically unhealthy phenotype while maintaining metabolic health during weight loss [109].
- Novel Adipokine-Based Therapies: Emerging approaches targeting adipose tissue function through adipokine supplementation or receptor modulation are under investigation. Recombinant adiponectin, adiponectin receptor agonists, and agents targeting the newly discovered adipokine family of C1q/TNF-related proteins (CTRPs) have shown promising metabolic effects in preclinical studies.

These pharmacological approaches highlight the potential for targeting specific pathophysiological mechanisms underlying metabolic health in obesity rather than focusing solely on weight reduction. However, long-term safety and efficacy data remain limited for many of these approaches.

## 8. Clinical Implications and Risk Stratification

Given the evidence for both increased disease risk and temporal instability of MHO, translating research findings into practical clinical approaches becomes essential. This section examines how MHO concepts can be integrated into routine clinical practice.

### 8.1. Risk Stratification Tools

Body-fat percentage (%BF): Although not a substitute for metabolic profiling, DXA- or BIA-derived %BF provides useful context when BMI under- or over-estimates adiposity. A higher %BF at a given BMI associates with insulin resistance and dyslipidemia, whereas a lower %BF is characteristic of MHL/MHNW [5,50]. Given population and sex differences, %BF should be interpreted alongside the waist–hip ratio and TG/HDL-C rather than using fixed universal cut-points [5,13,14,110].

Visceral adipose tissue (VAT): Visceral fat burden is a key discriminator between MHO and MUO and independently tracks with hepatic steatosis, insulin resistance, and cardiovascular risk [12,52,53,54]. When available, MRI or CT quantification of VAT can refine risk beyond BMI and waist circumference; in clinical practice, surrogates such as the waist–hip ratio (Zembic criteria) and TyG index can partially capture this risk [14,110]. Integration with liver fat assessment (e.g., VCTE, MRE, or non-contrast MRI with AI-assisted staging) enhances risk prediction for fibrosis and cardiometabolic progression [111,112,113,114].

Pragmatic panel: For clinics without advanced imaging, a pragmatic panel combining the waist–hip ratio, TG/HDL-C, TyG index, and steatosis scores (e.g., Fatty Liver Index) can identify higher-risk MHO warranting closer follow-up or earlier pharmacologic therapy [6,14,110].

Several practical tools have been developed to identify MHO and stratify obesity-related risks beyond simple BMI assessment:

- Simplified MHO Criteria: Zembic et al. proposed a simplified clinical definition of MHO (systolic blood pressure < 130 mmHg, waist–hip ratio < 0.95 for women or <1.03 for men, and absence of diabetes) that outperformed traditional metabolic syndrome criteria in predicting cardiovascular outcomes [14]. This definition has been validated in multiple cohorts including the UK Biobank, Flemengho, and Hortega studies.
- Metabolic-BMI: This composite measure incorporates both BMI and metabolic factors into a single risk score, potentially providing more nuanced risk assessment than either measure alone.
- Liver Fat Indices: Recent developments in 2024 include enhanced diagnostic accuracy through combination approaches. Non-invasive assessments, including vibration-controlled transient elastography (VCTE), magnetic resonance elastography (MRE), and serum biomarkers, have high accuracy to diagnose advanced fibrosis and cirrhosis [111]. The newly developed LiverPRO algorithm, which obtained European CE approval in 2024, reliably identifies clinically significant liver fibrosis and elevated liver stiffness, predicting the risk of liver-related events in primary care [112]. AI-powered models utilizing non-contrast MRI, including T1WI and T2FS, accurately stage liver fibrosis [113], and AI enhances diagnostic accuracy and efficiency, aiding clinicians in making more informed treatment decisions [114].
- Using multiparametric MRI to quantify liver fat content has been suggested as a more precise method for risk stratification when available [6]. This approach provides a direct measurement of a key determinant of metabolic health in obesity.
- Triglyceride–Glucose Index: This simple index combining fasting triglycerides and glucose levels has demonstrated utility for identifying insulin resistance and predicting transition from MHO to MUO [110].
- Novel Biomarkers: ITLN1 (Omentin-1), produced by specific mesothelial cell populations, has been identified as significantly higher in individuals with MHO compared to MUO [58]. This adipokine was exclusively expressed by mesothelial cells within visceral adipose tissue and was not present in subcutaneous adipose tissue. Plasma Omentin-1 levels were significantly higher in MHO compared to MUO, establishing it as a promising marker for visceral adipose tissue functionality. Additional biomarkers include circulating microRNAs (miR-122-5p, miR-151a-3p, miR-126-5p, and miR-21-5p) and point-of-care technologies integrating miR-34a-5p, YKL-40, and comprehensive metabolomic panels [25].

### 8.2. Clinical Monitoring Strategies

Given the risk of transition from MHO to MUO, regular monitoring of metabolic parameters in all individuals with obesity appears prudent. Baseline assessment should include the following:

1. Comprehensive metabolic panel including liver enzymes;
2. Lipid profile including triglyceride/HDL ratio;
3. Anthropometric measurements including waist-to-hip ratio;
4. Blood pressure assessment;
5. Screening for metabolic dysfunction-associated steatotic liver disease (ultrasonography or biomarkers);
6. Assessment of inflammatory markers (e.g., hsCRP) when available [115].

The optimal frequency of monitoring remains uncertain, though annual reassessment of metabolic status appears reasonable based on observed transition rates. More frequent monitoring may be appropriate for those with risk factors for transition to MUO, including older age, higher BMI or waist circumference, or elevated baseline liver fat [97].

### 8.3. Personalized Intervention Approaches

The MHO concept supports a more nuanced approach to obesity management beyond universal weight loss recommendations. Several strategies might be considered:

- MHO Maintenance: For metabolically healthy individuals with mild obesity and no other clinical indications for weight loss, interventions focused on maintaining metabolic health rather than aggressive weight reduction might be appropriate. Physical activity promotion, Mediterranean-style dietary patterns, and monitoring for transition to MUO could be emphasized.
- Targeting Specific Metabolic Parameters: For individuals with MHO with emerging metabolic abnormalities in specific domains (e.g., borderline elevated blood pressure or glucose), targeted interventions addressing these specific parameters might be prioritized over general weight loss.
- Aggressive Approach for High-Risk MHO: For individuals with MHO with significant risk factors for transition to MUO (e.g., elevated liver fat, family history of diabetes, and advancing age), more aggressive lifestyle and potentially pharmacological interventions may be warranted despite current metabolic health.
- A personalized treatment algorithm based on stratification of obesity phenotypes has been proposed [6]. While all individuals with obesity should receive basic lifestyle interventions, additional pharmacological treatments should be tailored to specific metabolic risk profiles, with particular attention to those showing early signs of transition from MHO to MUO.

The heterogeneity in metabolic phenotypes suggests that treatment strategies should be tailored to individual adipose tissue characteristics rather than based solely on BMI. Recent single-nucleus studies have shown that specific adipocyte subtypes have an inverse association with metabolic health state, and although BMI is clinically applicable, studies show that obesity defined by this metric is heterogeneous, with individuals having similar BMIs showing remarkable differences in health risk [58]. Detailed metabolic phenotyping of persons with obesity is invaluable in understanding pathophysiology and is needed to identify high-risk individuals, thereby paving the way for optimization of prevention and treatment strategies [116]. There is a growing “need to go beyond BMI” notion in clinical obesity medicine in the quest for better personalizing treatment, and assessment of adipose tissue health is certainly a potential avenue for decision making [117]. Multiple obesity phenotypes can coexist in the same individual, and post hoc analyses have shown that these obesity phenotypes predict weight loss response to anti-obesity medications and bariatric endoscopic devices [118].

## 9. Future Directions

While significant progress has been made in understanding MHO, numerous knowledge gaps remain. Key areas for future research include the following:

### 9.1. Standardized Definition and Classification

The development and validation of harmonized, clinically practical definitions of MHO represents a critical need. Future efforts should focus on definitions that incorporate emerging markers of metabolic health beyond traditional risk factors, potentially including the following:

- Non-invasive assessments of ectopic fat deposition (liver, pancreas, and heart);
- Markers of adipose tissue function and expandability;
- Novel biomarkers reflecting inflammatory status;
- Genetic and metabolomic profiles predicting long-term metabolic resilience.

The integration of these factors into clinical risk prediction models could substantially improve the identification of truly metabolically healthy obesity versus high-risk transitional states.

### 9.2. Mechanistic Insights

Further research is needed to elucidate the precise biological mechanisms underlying metabolic health in obesity. Specific priorities include the following:

- Systems biology approaches integrating genomic, transcriptomic, proteomic, and metabolomic data;
- Advanced adipose tissue phenotyping techniques examining depot-specific function;
- Investigation of gut microbiome contributions to metabolic health in obesity;
- Exploration of brain–adipose tissue communication pathways;
- Elucidation of sex-specific mechanisms explaining the higher prevalence of MHO in women.

Recent advances in single-cell and spatial transcriptomic analyses have opened new avenues for understanding the cellular and molecular mechanisms of MHO [58,59,60]. The identification of mesothelial cells as key drivers of metabolic health in visceral adipose tissue represents a novel direction worthy of further investigation. The finding that specific mesothelial populations undergo transition from epithelial to mesenchymal states correlating with metabolic health status provides a potential target for therapeutic interventions.

### 9.3. Longitudinal Natural History Studies

Comprehensive long-term studies tracking the natural history of MHO across diverse populations are needed to better understand the following:

- Predictors of maintained metabolic health versus transition to MUO;
- Critical time windows for intervention to prevent metabolic deterioration;
- Impact of life-stage transitions (puberty, pregnancy, and menopause) on MHO stability;
- Influence of aging on metabolic protection mechanisms.

The discovery of epigenetic memory of obesity provides molecular evidence for understanding long-term trajectories, with adipose tissue retaining “obesogenic memory” after weight loss that may explain yo-yo dieting effects [86].

### 9.4. Intervention Studies

Targeted intervention studies specifically addressing populations with MHO are largely lacking. Future research should focus on the following:

- Randomized trials comparing different lifestyle intervention strategies in MHO;
- Evaluation of pharmacological approaches targeting specific mechanisms underlying MHO;
- Studies examining the impact of various weight loss approaches on long-term outcomes in MHO;
- Determination of optimal monitoring and intervention thresholds for preventing transition to MUO.

Clinical trials specifically designed to assess interventions in the population with MHO are needed, as most previous studies have not stratified participants by metabolic phenotype [6]. Recent evidence suggests that concurrent training combining aerobic and resistance exercise may be particularly effective for individuals with MHO. Lentejas et al. (2024) found that concurrent training was more effective in reducing BMI, body fat composition, and CRP compared to aerobic and resistance exercises alone in metabolically healthy obese adults [67].

### 9.5. Novel Therapeutic Targets

The MHO phenotype provides a unique opportunity to identify novel therapeutic targets for obesity-related complications. Promising directions include the following:

- Approaches promoting adipose tissue expandability and healthy remodeling;
- Therapies targeting ectopic fat redistribution rather than total weight reduction;
- Interventions modulating adipose tissue immunometabolism;
- Treatments targeting specific adipokine signaling pathways identified in MHO.

Several potential targets have been identified [58], including mesothelial cell populations that could be manipulated to maintain or induce a metabolically healthy state. The finding that mesothelial cells might undergo a switch from a mesenchymal to an inflammatory phenotype under disease conditions suggests that interventions promoting the mesenchymal phenotype might improve metabolic health in obesity. The identification of CD9+CD55low adipose progenitor cells that impair glucose homeostasis through PEDF secretion provides another therapeutic target [59].

Research considering the impact of abdominal obesity (visceral fat accumulation) on MHO prognosis is particularly important for developing effective interventions.

## 10. Conclusions

The concept of metabolically healthy obesity has profoundly challenged the simplistic notion that excess adiposity invariably leads to metabolic dysfunction. While individuals with MHO exhibit a relatively favorable metabolic profile compared to their metabolically unhealthy counterparts, they are not entirely immune to increased health risks, particularly for type 2 diabetes, cardiovascular disease, chronic kidney disease, and certain cancers. The transient nature of MHO, with approximately 30–50% of individuals progressing to MUO within 4–10 years, emphasizes the importance of ongoing monitoring.

The ability to maintain metabolic health in the context of obesity is influenced by a complex interplay of beneficial adipose tissue distribution, enhanced adipocyte function, favorable genetic and epigenetic profiles, and healthy lifestyle choices. Recent advances in single-cell transcriptomics have provided unprecedented insights into the cellular determinants of healthy adipose tissue expansion, identifying mesothelial cells, specific adipose progenitor cell populations, and their secreted factors as key regulators of metabolic health.

The discovery of persistent epigenetic memory of obesity provides molecular evidence for why MHO often represents a transient rather than stable state. Emerging therapeutic approaches, particularly dual and triple incretin agonists, show promise for maintaining metabolic health during weight loss, though long-term outcomes remain to be determined.

The recognition of MHO offers an opportunity for more precise risk stratification and the development of personalized prevention and treatment strategies for obesity. Future research should focus on refining MHO definitions incorporating hepatic steatosis assessment, conducting comprehensive longitudinal studies, elucidating underlying mechanisms through advanced molecular techniques, and developing targeted interventions to preserve metabolic health in this unique and challenging obesity phenotype. As our understanding evolves, the integration of genetic, epigenetic, and lifestyle factors will be crucial for developing precision medicine approaches that can effectively prevent the transition from MHO to metabolically unhealthy states and reduce the overall burden of obesity-related diseases.

## Data Availability

Not applicable.

## Abbreviations

The following abbreviations are used in this manuscript:

BMI	Body mass index
DOI	Digital object identifier
IRS	Insulin receptor substrate
MASLD	Metabolic dysfunction-associated steatotic liver disease
MHNW	Metabolically healthy normal weight
MHO	Metabolically healthy obesity
MUO	Metabolically unhealthy obesity
NAFLD	Non-alcoholic fatty liver disease
